# Developing effective communication materials on the health effects of climate change for vulnerable groups: a mixed methods study

**DOI:** 10.1186/s12889-016-3546-3

**Published:** 2016-09-07

**Authors:** Jennifer M. Kreslake, Katherine M. Price, Mona Sarfaty

**Affiliations:** Program on Climate & Health, Center for Climate Change Communication, George Mason University, 4400 University Drive, MS 6A8, Fairfax, VA 22030 USA

**Keywords:** Climate change, Global warming, Health impacts, Health disparities, Health literacy, Health communication, Chronic disease management

## Abstract

**Background:**

Individuals with chronic health conditions or low socioeconomic status (SES) are more vulnerable to the health impacts of climate change. Health communication can provide information on the management of these impacts. This study tested, among vulnerable audiences, whether viewing targeted materials increases knowledge about the health impacts of climate change and strength of climate change beliefs, and whether each are associated with stronger intentions to practice recommended behaviors.

**Methods:**

Low-SES respondents with chronic conditions were recruited for an online survey in six cities. Respondents were shown targeted materials illustrating the relationship between climate change and chronic conditions. Changes in knowledge and climate change beliefs (pre- and post-test) and behavioral intentions (post-test only) were tested using McNemar tests of marginal frequencies of two binary outcomes or paired t-tests, and multivariable linear regression. Qualitative interviews were conducted among target audiences to triangulate survey findings and make recommendations on the design of messages.

**Results:**

Respondents (*N* = 122) reflected the target population regarding income, educational level and prevalence of household health conditions. (1) Knowledge. Significant increases in knowledge were found regarding: groups that are most vulnerable to heat (children [*p* < 0.001], individuals with heart disease [*p* < 0.001], or lung disease [*p* = 0.019]); and environmental conditions that increase allergy-producing pollen (increased heat [*p* = 0.003], increased carbon dioxide [*p* < 0.001]). (2) Strength of certainty that climate change is happening increased significantly between pre- and post-test (*p* < 0.001), as did belief that climate change affected respondents’ health (*p* < 0.001). (3) Behavioral intention. At post-test, higher knowledge of heat vulnerabilities and environmental conditions that trigger pollen allergies were associated with greater behavioral intention scores (*p* = 0.001 and *p* = 0.002, respectively). In-depth interviews (*N* = 15) revealed that vulnerable audiences are interested in immediate-term advice on health management and protective behaviors related to their chronic conditions, but took less notice of messages about collective action to slow or stop climate change. Respondents identified both appealing and less favorable design elements in the materials.

**Conclusions:**

Individuals who are vulnerable to the health effects of climate change benefit from communication materials that explain, using graphics and concise language, how climate change affects health conditions and how to engage in protective adaptation behaviors.

## Background

Climate change is already occurring, and its impacts are projected to increase into the next century and beyond [1]. Environmental conditions that will contribute to greater human morbidity and mortality from climate change include increases in air pollution, intense storms and extreme heat [2, 3]. Greater levels of particulate air pollution and ground-level ozone exacerbate respiratory conditions, resulting in increased hospital admissions, emergency room visits and mortality [4, 5]. Climate change will increase pollen production because of more frost-free days, warmer seasonal temperatures and longer growing seasons due to an abundance of carbon dioxide, and the resulting pollen from allergenic plant species contributes to allergies and asthma [6, 7]. Extreme heat contributes to hospital admissions and death for those with cardiovascular, cerebrovascular, kidney and respiratory disorders [8, 9]. Chronic disease can limit mobility during extreme weather events, and extreme weather disrupts access to routine medical care [10].

Populations that are especially vulnerable to these health impacts include those with chronic health conditions, low socioeconomic status (SES), children, the elderly and some racial/ethnic minority groups [2, 11]. At-risk populations can have co-occurring vulnerabilities which increase risk for illness, injury or death from environmental conditions attributable to climate change, particularly if they are simultaneously lacking the financial, social or community resilience necessary to cope with or recover from additional stressors [12].

Previous research demonstrates that messages designed to raise awareness of health risks from weather events can reduce morbidity and mortality among vulnerable populations. These findings may be applicable to the extreme weather and environmental conditions that are projected to increase due to climate change. For example, the implementation of warning systems in France after devastating heat waves in 2003 reduced morbidity and mortality in vulnerable groups during subsequent heat waves by 2006 [13]. Hurricane evacuation notices have been shown to increase the likelihood of evacuation among coastal residents when disseminated by public officials [14].

Age, gender, race/ethnicity and socioeconomic status are associated with differences in perceived risk of natural disasters, as well as how individuals obtain and react to information about natural disasters, extreme weather and environmental conditions [12]. Previous research on broadcast warnings about extreme weather events and natural disasters identifies target audiences by demographic characteristics that are associated with increased vulnerability to health impacts (e.g., age, race/ethnicity) [12, 15]. Delivery channels for these messages have included internet-based interventions, media sources (television, radio, newspaper) and printed materials [11, 15]. Qualitative research among residents of cities with racial/ethnic- and SES-related disparities in heat-related morbidity and mortality described how cooling practices during heat waves may vary according to differences in risk perceptions, resources and social norms [16]. Another qualitative study conducted among low-SES racial and ethnic minorities observed that respondents were especially attuned to the effects of local, acute environmental health risks (e.g., sanitation) [17].

Health communication materials have potential to improve knowledge, attitudes and protective behavior related to climate change and health among vulnerable populations [18, 19]. Previous research provides guidance on message development to improve accessibility to a variety of audiences. Design features that address literacy, visual, and auditory limitations include a variety of text or visual aids (e.g., symbols, images), large print and/or Braille [15]. Other aspects to consider are language and culture of the intended audience [18, 20]. Taylor-Clarke et al. [17] suggest the use of concise materials, designed to reduce frustration in navigating an abundance of information, that also address potential inequities in Internet access.

To our knowledge, no study has tested the effectiveness of communication materials that target vulnerable populations and describe the health impacts of climate change due to weather and environmental conditions that are projected to increase in intensity and frequency. This study defines vulnerable individuals as having at least one of the chronic health condition(s) projected to be impacted by climate change present in the household (respiratory illness including asthma, pollen allergies, heart disease, obesity or diabetes) and low socioeconomic status as defined by income and educational level [2, 3].

This mixed-methods study was designed to test whether: (1) viewing targeted educational materials increases knowledge about health effects of climate change among vulnerable groups; (2) greater knowledge of these health effects is associated with stronger intentions to practice recommended behaviors toward climate change preparedness (“adaptation”) and prevention (“mitigation”); and (3) viewing targeted educational materials strengthens certainty that climate change is happening and that it impacts health.

## Methods

### Study design

This study tested the effectiveness of health communication materials to inform their eventual use in community-based settings using a quasi-experimental pre-post study design. An online survey assessed within-subject changes in knowledge, beliefs and behavioral intentions before and after viewing study materials. The findings of the online survey are supplemented by in-depth interviews among a small number of target audience members to obtain greater insight into their interpretation of the study materials.

### Study materials

Study materials were designed by a public health research and advocacy organization; the authors of this study were not involved in content creation. Materials were designed to pictorially represent the mechanisms by which climate change – specifically, weather events and environmental conditions projected to be affected by climate change – affects chronic health conditions [2]. Recommendations for protective action are consistent with the scientific literature concerning approaches to climate change in clinical practice [21–23]. Five multicolored illustrations described how climate change impacts health conditions (asthma, allergies, heart disease, obesity and the health effects of extreme heat). The Flesch-Kincaid Grade Level for descriptive text within the illustrations was 7.8 [24].

Materials were designed to be poster-sized. Table 1 summarizes content and design features of the posters. A panel at the bottom of each poster provided lists of what individuals can do to protect their health from the specific condition (“Healthy You”), individual actions toward climate change mitigation such as walking, biking or taking public transit instead of driving (“Healthy Places”), and what respondents could do to engage in collective action for climate change mitigation, such as supporting policies to limit carbon pollution or support clean energy (“Healthy Planet”).

**Table 1:** Key design features and content included in study materials

Details about one health condition affected by climate change
• Asthma • Allergies • Health effects of extreme heat • Obesity/food systems • Heart disease
Human figure icons in conversation (via speech bubbles) about relationship between climate change and health conditions
Mechanisms of climate change, explained at 8^th^-grade readability level or lower
• “Carbon pollution makes the world warmer and changes our climate.” • “Climate change will lead to more extreme heat events.” • “Higher temperatures mean spring comes earlier and allergy season lasts longer… A longer season means more pollen. Carbon dioxide in the atmosphere is like food for plants, helping them grow bigger and make more pollen.” • “Extreme heat can make working outdoors dangerous.” • “Extreme heat can lead to irregular heartbeats and stroke.” • “Air pollution increases the risk of heart attacks.” • “Climate change leads to drought, heat and extreme rain, making it harder to grow crops and raise animals for food.” • Red meat is high in saturated fats and can increase the risk of heart disease. Processed food high in sugars, salt and saturated fats can increase obesity, diabetes and heart disease.” • “Red meat and processed foods use more energy and more chemical fertilizers than local fresh vegetables and fruits. Using more energy releases more global warming pollution.”
Recommended actions
• Individual protective or preventive behaviors toward climate change adaptation (“Healthy You”) ○ Stay hydrated, stay cool in hot weather ○ Use local information resources (weather reports, Air Quality Index, daily pollen reports) ○ Reduce exposure (to pollen, heat, smog) ○ Monitor family members, friends and neighbors during hot weather ○ Eat less red meat and processed foods • Individual, local actions toward climate change mitigation (“Healthy Places”) ○ Ride bikes, walk or use public transportation ○ Eat healthy, local foods ○ Plant trees that produce low levels of pollen ○ Discover ways to use less energy • Collective action toward climate change mitigation (“Healthy Planet”) ○ Support actions to limit carbon pollution, clean energy, local advocacy for climate action

### Online survey

#### Data collection

Pre-test and post-test data were collected in a single online survey session. The online survey was administered during June/July of 2015. Respondents were recruited from online community bulletin boards in select metropolitan areas using a nonprobability purposive sampling design. Metropolitan regions were selected for relatively poor air quality, racial/ethnic diversity and high proportion of low-income residents. Metropolitan areas (with estimated population sizes in 2014) were: Los Angeles, CA (13.3 million); Houston, TX (6.5 million); Philadelphia, PA (6.1 million); Atlanta, GA (5.6 million); Detroit, MI (4.3 million); and Cleveland, OH (2.1 million) [25–28]. Multiple geographically dispersed metropolitan areas were sampled to ensure variability across multiple climate regions in the United States, but was not done with the intention of drawing conclusions about regional differences between respondents. The largest number of respondents was from California (*n* = 38, 33 % of the sample), followed by Pennsylvania (*n* = 35, 30 %), Texas (*n* = 20, 17 %), Georgia (*n* = 12, 10.4 %), and Midwestern states (Ohio or Michigan, *n* = 10, 8.7 %). Due to the small number of respondents per city, respondents cannot be considered representative of households in each geographic region. To address concerns about validity of participants’ location, the survey software screened respondents’ Internet Protocol (IP) addresses to ensure that physical location corresponded with the sampled geographic region.

The recruitment announcement invited participants with select chronic conditions (asthma, allergies, heart disease, obesity and diabetes) in the household to determine their eligibility for an online survey. Respondents to the online recruitment advertisement took a brief screening survey that screened according to socioeconomic status (those with an annual household income lower than $40,000 and educational attainment lower than a 4-year college degree met inclusion criteria). The $40,000 income criterion was selected to provide an initial approximation of households living below “reasonable comfort level” and to exclude high-income respondents [29]. “Low income” has been defined elsewhere as twice the federal poverty level, determined as a function of household income and family size (for example, in 2015, a low-income household of three people earning less than $40,180.00 is at 200 % of the federal poverty level) [30, 31]. It was not possible to screen on the basis of this formula due to technological constraints, but restricting the household income to $40,000 ensured that regardless of family size, we were almost certainly preventing “high income” respondents from participating. Data was also collected on household size. Post hoc analysis of the demographic data based on family size and household income revealed that most of the sample (82 %) fell at or below 200 % of the federal poverty guidelines. The two most affluent respondents in our sample each lived in a single-person household with an income between $31,000 and $40,000.

The study materials were administered to respondents using an onscreen version of the posters within an online survey interface. Each respondent was shown a total of five posters in randomized order.

The survey measured changes in knowledge of environmental contributors to health symptoms, and climate change beliefs, before viewing the study materials (pre-test) and after (post-test). At pre-test, respondents were asked what sources they used for information about their health conditions, and reported any environmental conditions that made their existing health conditions worse (i.e., air pollution/smog, hot weather, humidity, pollen or none of these). Questions on respondents’ anticipated use of the information provided by study materials and intentions to practice recommended behaviors were asked at post-test.

#### Measures at pre-test only

##### Household health status

Respondents selected from a list of chronic health conditions that affected members of their household (asthma, allergies, obesity, diabetes, heart disease or health effects from extreme heat). Response categories were not mutually exclusive. A subsequent item asked which household member(s) were affected by the selected conditions (self, partner/spouse, parent, other adults in household, children [age 0–12 or 13–17]). Respondents were asked to identify environmental factors that worsened their health condition(s): air pollution/smog; hot weather; humidity; pollen; or none.

##### Sources of information for chronic disease management

Respondents reported where they received information about treating the health condition(s) that affected their household member(s): doctor’s office/health clinic; hospital emergency room; pharmacy; children’s school; the Internet; friends or family members; other sources (open-ended); or none of these. Response categories were not mutually exclusive.

#### Pre- and post-test measures

##### Knowledge of environmental health effects

Three items measured knowledge about environmental triggers of health conditions and vulnerable populations. Respondents were asked to identify the following: (1) Populations that are vulnerable to extreme heat (“Extremely hot weather is especially hard for some people. What are some groups of people that you think would be more affected by heat?”). Response options were: older adults; people with heart disease; people with lung disease; young children; people without air conditioning; other (open-ended); none; and an incorrect “sham” response (people with sensitive skin). (2) Triggers of asthma (“Asthma is a lung condition that makes it difficult to breathe. Do you know of any causes that can make asthma worse for people with this condition?”). Response options included: smoke; pollen; pet dander; air pollution/smog; other (open-ended); none of these; and an incorrect “sham” response (certain foods, such as peanuts). (3) Triggers of pollen allergies (“Allergies can affect people who are sensitive to pollen. Do you know of any causes that can make allergies worse for people with this condition?”). Response options were: weeds, grasses and trees that produce pollen; longer growing season for weeds, grasses and trees; heat; carbon dioxide; other (open-ended); none; and an incorrect “sham” response (lightning). Response categories were not mutually exclusive.

##### Strength of climate change belief certainty

Respondents were presented with a statement defining climate change (“Climate change refers to the idea that the world’s average temperature has been increasing for the past 150 years, may be increasing in the future, and that the world’s climate is changing as a result.”). A series of questions measured respondents’ strength of certainty that climate change is happening. Responses were coded on a nine-point index ranging from 1 (extremely unsure) to 9 (extremely sure), with “5” as a neutral midpoint (“don’t know”).

##### Belief that climate change affects health

Respondents were asked whether climate change affected any health conditions experienced by the respondent or their household members (yes/no/don’t know).

#### Measures at post-test only

##### Delivery channels

Immediately following the presentation of study materials, respondents were asked to identify the most useful channels for messages. Response options included: poster in a doctor’s office; pamphlet (handout) in a doctor’s office; children’s schools; websites; in discussion with doctor; nowhere; and other (open-ended). Response categories were not mutually exclusive.

##### Intentions to engage in recommended behaviors

Intentions to engage in 11 recommended behaviors within a specific time frame (the next 3 months or, when relevant, in the next election) were measured using a seven-point scale. Anchor points were “extremely unlikely” (1) and “extremely likely” (7) [32, 33]. Recommended behaviors included individual protective behaviors for adaptation to climate change (e.g., encouraging family members to drink water and stay out of the sun during extremely hot weather; using the Air Quality Index to guide outdoor activities); individual behaviors toward climate change mitigation (e.g., reducing energy use in their home); or collective action toward climate change mitigation (voting for laws that limit pollution or require clean energy).

#### Statistical analysis

##### Change in knowledge and beliefs between pre- and post-test

McNemar tests of marginal frequencies of two binary outcomes were used to test whether respondents differed in their knowledge of each heat-vulnerable group, allergy triggers, and asthma triggers between pre- and post-test. A paired *t*-test was used to assess significant changes in certainty that climate change is happening.

##### Association between knowledge and behavioral intention

The number of correct responses to each knowledge question at post-test was summed into a score. Knowledge scores for identifying heat-vulnerable populations ranged from 0 to 5. Knowledge scores for identifying asthma triggers ranged from 0 to 4. Knowledge scores for identifying allergy triggers ranged from 0 to 4.

Respondent intention ratings for each recommended behavior were combined into an aggregated index (Chronbach’s α = 0.90). Respondents who selected an extreme value on all 11 behaviors (*n* = 13) were omitted.

Three multivariable linear regression models were used to test the association between each knowledge score at post-test (the independent variable) and the behavioral intention index (the dependent variable). Covariates were race/ethnicity, state of residence, post-test certainty that climate change is happening, post-test belief that climate change affects their health (recoded into a dichotomous variable for statistical analysis; 1 = yes and 0 = no/don’t know), and political orientation (1 = very liberal to 5 = very conservative).

### In-depth interviews

#### Data collection

In-depth interviews were conducted among respondents with low SES in an urban center accessible by public transportation (Washington, DC, selected for its proximity to the researchers). Respondents were recruited through an online community bulletin board. The recruitment announcement invited participants with select chronic conditions (asthma, allergies, heart disease, obesity and diabetes) in the household to determine their eligibility for an in-person discussion as part of a research study. Respondents to the online recruitment advertisement took a brief screening survey that identified eligible respondents (those with an annual household income lower than $40,000 and educational attainment lower than a 4-year college degree).

Interviews were conducted until saturation was achieved (*N* = 11) [34]. Each interview took approximately 30 min to complete. A semi-structured interview guide was designed to elicit participants’ extemporaneous understanding of climate change and its health impacts, their perceptions of the study materials, and their recommendations to improve the design, layout, content and language of the study materials.

In the interest of time and to minimize burden to subjects, participants were each shown three of the five posters on a randomly assigned rotation. Besides differing according to the emphasis on various health conditions, some content was repeated across posters. Each poster was viewed by at least three respondents apiece. Posters were printed half-sized and mounted on foam backing. The posters were discussed individually, and then compared at the end of the interview. Study participants were asked to describe the poster content in their own words, discuss what they found most or least interesting and why, how they would use the information on the posters (if at all), and if they had recommendations for poster content or design. One of the study authors conducted the interviews with a note taker present. Interviews were audio recorded and transcribed for qualitative analysis.

#### Qualitative analysis

A coding scheme was developed using an inductive approach to analysis of the in-depth interviews. Two researchers served as primary coders and a third coder resolved any disagreement. Codes were developed and organized according to emergent themes (Table 2).

**Table 2:** Coding scheme for inductive analysis of in-depth interviews (*N* = 11)

	Number of mentions
Themes (with associated codes)	
Weather is better understood than climate change	
Personal experience/anecdotal evidence	27
Hot weather/heat: immediate concerns	23
Pollution: disconnected from weather	6
General understanding of climate change	1
Weather conditions (not climate change)	1
Confusion/misinterpretation/unintended consequences	
Dietary advice/advice to lose weight (disconnected from environmental impact, food systems or climate change)	28
Demonstrated gaps in existing knowledge	27
Confusion about meaning	16
Poster offers impractical advice	10
Advice to engage in harmful activity (e.g., advice to engage in walking/biking on poster about heat effects)	6
Health education strategies	
Activating existing knowledge	18
Intended audience/who should see	17
Suggested locations/venues to display	13
Health vulnerabilities	13
Not enough information provided	11
Appeal/liking	8
Recognition of self as audience	3
Behavioral modeling	3
Lack of available education on topic(s)	3
Poor populations	1
Shock/fear appeals	1
Attitudes and beliefs about climate change and health	
Explains mechanisms of climate change effects on health	17
Own perceptions/explanation of systemic issues	17
Learned something/“aha moment”	11
Emphasis on health (not climate change)	9
Individual behaviors	8
Perceived ignorance of others	7
Environmental triggers or causes of symptoms	7
Apathy about climate change/health	5
Avoidance/denial about climate change/health	4
Policy/government	4
Collective action	2
Clean energy/low pollution	2
Design	
Organization/layout	20
Appealing elements	14
Replace cartoons with photos of human faces/figures	9
Feature humans experiencing health effects	9
Suggestions for design	9
Suggestions for content	9
Color	8
Cartoons: appropriate for some	8
Cartoons: off-putting	6
Health literacy concerns	5
Cluttered/too much text	1

## Results

### Online survey

#### Sample description

Online survey respondents closely resembled the target audience for the study materials due to inclusion criteria (Table 3). The highest level of education completed by this sample was some college (or a 2-year degree) (76.7 %). The majority of respondents (64.4 %) had an income between $31,000 and $40,000 per year. The remainder of the sample had lower levels of education or lower income. Over one-quarter of the sample was Black (28.8 %) and a similar proportion was White (27.1 %). Hispanic respondents comprised 17.8 % of the sample. Most respondents were between 25 and 44 years old (73.3 %). The sample was mostly female (71.9 %).

**Table 3:** Online survey respondent demographics and household health status (*N* = 122)

	Number	Percent
Race/Ethnicity	(*n* = 118)	
White	32	27.1
Black	34	28.8
Hispanic	21	17.8
Other	31	26.3
Age	(*n* = 120)	
18–24	18	15.0
25–34	49	40.8
35–44	39	32.5
45 and above	14	11.7
Gender	(*n* = 122)	
Female	88	72.1
Male	34	27.9
Education	(*n* = 120)	
Some high school	8	6.7
High school or GED	20	16.7
Some college/2-year degree	92	76.7
Income	(*n* = 118)	
Less than 20 K	18	15.3
20 K–30 K	24	20.3
31 K–40 K	75	64.4
State of Residence	(*n* = 115)	
California	38	33.0
Texas	20	17.4
Pennsylvania	35	30.4
Georgia	12	10.4
Ohio or Michigan (Midwest)	10	8.7
Household Health Conditions	(*n* = 117)	
Asthma	69	59.0
Pollen Allergies	65	56.0
Obesity	33	28.5
Diabetes	17	14.7
Health effects from extreme heat	21	18.1
Affected household members	(*n* = 116)	
Self	81	69.8
Partner	28	24.1
Parent	37	31.9
Other adult household member	4	3.5
Child	12	10.3

Approximately half of respondents reported that they had children living in the household (53 %); one-third did not (35 %), and an additional 12 % declined to answer. The average number of members in the respondents’ household was 3.3 (range: 1–7, SD: 1.32).

Approximately half of the respondents had, or lived with a household member who had, asthma (59.0 %) and/or allergies (56.0 %). Respondents also reported obesity (28.5 %), health effects from hot weather (18.1 %), coronary heart disease or other heart conditions (16.4 %), and/or diabetes (14.7 %) being present in their household. Nearly half of households had two chronic conditions (45.1 %), one-third had one condition (30.3 %), and 17.2 % had three or more chronic conditions. Respondents reported that these health conditions were affecting themselves (69.8 %), their partner or spouse (24.1 %), a parent (31.9 %), their children (9.5 %), or another adult in their household (3.5 %).

Prior to viewing study materials, respondents identified triggers of existing health conditions in their household. The majority said their conditions were worsened by air pollution (75.0 %), hot weather (69.8 %), pollen (57.8 %), and humidity (47.4 %). The majority of respondents receive information about their respective health conditions from their doctor’s office or health clinic (>95 % for any identified health condition). A large proportion of those with heart conditions receive information from the emergency department (68.4 %). The emergency department is also used as a source of information by those with asthma (44.9 %), allergies (42.2 %), and heat sensitivity (52.4 %). It is unknown whether information is obtained during visits to the emergency department for acute health crises, or if the emergency department is a source of routine care for these respondents. The Internet was used as an information source by approximately half of the sample (56.9 %). Nearly half of respondents who suffered from heat effects used family and friends as sources of health information (42.9 %), while substantially fewer of those who had heart conditions (10.5 %) did so.

#### Change in knowledge and beliefs

After viewing the study materials, respondents demonstrated significant improvement in their knowledge about certain health effects of environmental conditions that are projected to increase due to climate change, and greater certainty that climate change is happening.

##### Knowledge of heat-vulnerable populations

**Fig. 1:**
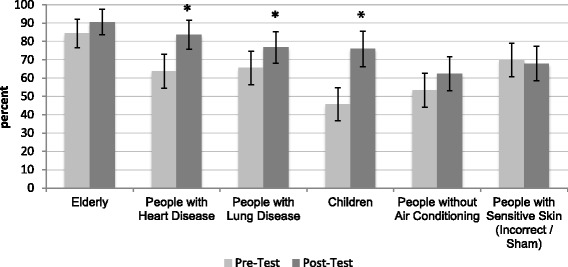
Proportion of respondents (95 % CI) who correctly identified populations that are vulnerable to health effects from extreme heat at pre- and post-test (*n* = 116)

At pre-test, the majority of respondents were aware that the elderly (*n* = 98, 84.5 %), people with lung diseases (*n* = 76, 65.5 %), and people with heart disease (*n* = 74, 63.8 %) were more affected by heat than the general population. Close to half were aware that children (*n* = 53, 45.7 %) and people without air conditioning (*n* = 62, 53.4 %) were differentially affected. By post-test, a greater proportion of respondents correctly identified people with heart disease (*n* = 97, 83.6 %) or lung disease (*n* = 89, 76.7 %), children (*n* = 87, 75.0 %), and people without air conditioning (*n* = 73, 62.4 %) as being vulnerable to heat effects. This increase was statistically significant for heart disease (McNemar’s *χ*^2^ = 14.30, *p* < 0.001), lung disease (McNemar’s *χ*^2^ = 6.26, *p* = 0.019), and children (McNemar’s *χ*^2^ = 24.08, *p* < 0.001) (Fig. 1).

##### Knowledge of allergy triggers

**Fig. 2:**
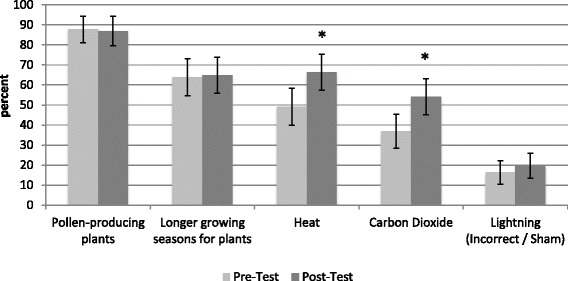
Proportion of respondents (95 % CI) who correctly identified environmental conditions that worsen allergies at pre- and post-test (*n* = 116)

Knowledge of environmental triggers for allergy sufferers improved significantly for abstract concepts related to plant growth and allergen production (heat, carbon dioxide) after viewing the study materials (Fig. 2). At pre-test, 52.6 % of respondents (*n* = 61) identified heat as a contributor to worsening allergies; 69.8 % (*n* = 81) selected heat at post-test. At pre-test, only 38.8 % of respondents (*n* = 45) identified carbon dioxide as a contributor to allergies, 56.9 % (*n* = 66) did so at post-test. The increases were statistically significant for heat (McNemar’s *χ*^2^ = 9.52, *p* = 0.003) and carbon dioxide (McNemar’s *χ*^2^ = 12.60, *p* < 0.001). Respondents were largely aware of other pollen allergy triggers before viewing the study materials, and these levels were sustained (92.2 % [*n* = 107] identified pollen-producing plants at pre-test, 91.4 % [*n* = 106] at post-test; 66.4 % [*n* = 77] identified longer growing seasons for plants at pre-test, 68.1 % [*n* = 79] did so at post-test).

##### Knowledge of asthma triggers

Knowledge of environmental exposures that trigger asthma attacks was high at the beginning of the survey (over half of the sample had a household member, including themselves, who suffered from asthma), but did not significantly improve (and for some items, declined slightly) after viewing the study materials. Although smoke was identified in the asthma study materials as a trigger, fewer respondents correctly identified it after viewing the study materials (93.1 % [*n* = 108] at pre-test, 86.2 % [*n* = 100] at post-test) the majority of respondents correctly identified pollen at pre-test (81.9 %, *n* = 95) and post-test (78.4 %, *n* = 91). Pet dander was identified by less than half the sample (46.6 % at pre-test [*n* = 54], 41.4 % at post-test [*n* = 48]) as a trigger. Three-quarters of the sample correctly identified air pollution (or smog) as an asthma trigger before (76.7 %, *n* = 89) and after (78.4 %, *n* = 91) viewing the study materials. Significantly fewer respondents chose the incorrect response option (“certain foods, such as peanuts”) after viewing the study materials (42.2 % at pre-test [*n* = 49], 29.5 % at post-test [*n* = 31]; McNemar’s *χ*^2^ = 6.43, *p* = 0.017).

##### Climate change belief certainty

**Fig. 3:**
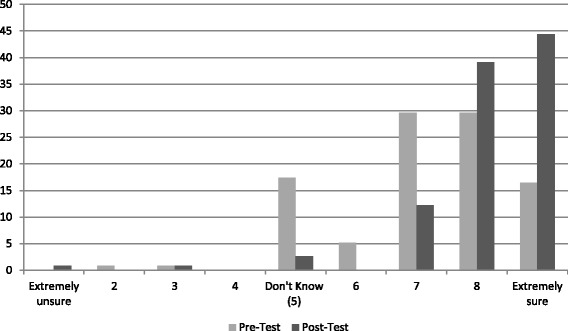
Proportion of respondents reporting strength of certainty that climate change is happening (*n* = 115)

Respondents demonstrated significantly greater certainty that climate change was happening at follow-up compared to baseline (Fig. 3). The mean score for climate change belief certainty was 7.15 (SE: 0.13) at pre-test, compared to 8.14 (SE: 0.11) at post-test (*p* < 0.001).

##### Belief that climate change affects health

**Fig. 4:**
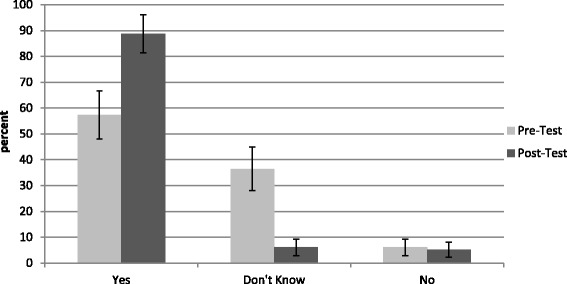
Proportion of respondents reporting belief that climate change is affecting the health of themselves or their households at pre- and post-test (*n* = 116)

Respondents had greater likelihood of reporting that climate change was affecting their health or household members’ health at post-test compared to pre-test (McNemar’s *χ*^2^ = 28.17, *p* < 0.001). At pre-test, over half (57.0 %, *n* = 65) believed that climate change affected their health, approximately one-third (36.8 %, *n* = 42) did not know, and a small proportion (6.1 %, *n* = 7) did not believe it affected their health. At post-test, the vast majority (88.7 %, *n* = 102) believed climate change affected their health, while a small proportion did not know (6.1 %, *n* = 7) or responded no (5.2 %, *n* = 6) (Fig. 4).

#### Predictors of behavioral intention

Greater knowledge at post-test was positively associated with intentions to practice recommended behaviors, controlling for covariates (Table 4). A higher knowledge score for identifying heat-vulnerable populations was associated with a higher behavioral intention index score (unstandardized *b*: 0.28, *p* = 0.001). A higher knowledge score for pollen allergy triggers was significantly associated with a higher behavioral intention index score (*b*: 0.26, *p* = 0.002), controlling for other covariates. A higher knowledge score for asthma was not significantly associated with the behavioral index.

**Table 4:** Predictors of intentions to practice recommended behaviors. (all measures at post-test) (*n* = 94)

	Behavioral intention index score	
	*b* (SE)	β
Knowledge of heat-vulnerable populations (summary score)	0.28 (0.08)**	0.37
Strength of certainty about climate change	0.09 (0.07)	0.13
Belief that climate change affects health of self/household	0.52 (0.27)	0.19
Political orientation (ref: very liberal)	0.10 (0.07)	0.13
Race/ethnicity (ref: White)		
Black	0.55 (0.25)*	0.29
Hispanic	0.77 (0.28)*	0.35
Other	0.19 (0.29)	0.10
State of residence (ref: California)		
Texas	−0.20 (0.26)	−0.09
Pennsylvania	−0.03 (0.23)	−0.01
Georgia	−0.22 (0.30)	−0.08
Ohio or Michigan (Midwest)	−0.76 (0.32)*	−0.27
	*R* ^2^ = 0.352	
	*F* (11,82) = 4.05**	
Knowledge of pollen allergy triggers (summary score)	0.26 (0.08)*	0.31
Strength of certainty about climate change	0.07 (0.07)	0.10
Belief that climate change affects health of self/household	0.57 (0.27)	0.21
Political orientation (ref: very liberal)	0.11 (0.07)	0.15
Race/ethnicity (ref: White)		
Black	0.46 (0.25)	0.24
Hispanic	0.51 (0.28)	0.23
Other	−0.09 (0.28)	−0.05
State of residence (ref: California)		
Texas	−0.18 (0.26)	−0.08
Pennsylvania	−0.04 (0.23)	−0.02
Georgia	−0.18 (0.30)	−0.06
Ohio or Michigan (Midwest)	−0.81 (0.32)*	−0.28
	*R* ^2^ = 0.337	
	*F* (11,82) = 3.80**	
Knowledge of asthma triggers (summary score)	0.10 (0.10)	0.11
Strength of certainty about climate change	0.09 (0.08)	0.12
Belief that climate change affects health of self/household	0.60 (0.29)*	0.22
Political orientation (ref: very liberal)	0.13 (0.07)	0.17
Race/ethnicity (ref: White)		
Black	0.50 (0.27)	0.26
Hispanic	0.44 (0.29)	0.20
Other	−0.08 (0.31)	−0.04
State of residence (ref: California)		
Texas	−0.31 (0.28)	−0.15
Pennsylvania	−0.17 (0.24)	−0.09
Georgia	−0.38 (0.32)	−0.14
Ohio or Michigan (Midwest)	−0.83 (0.34)	−0.29
	*R* ^2^ = 0.26	
	*F* (11,82) = 2.67**	

*p ≤ 0.05; **p ≤ 0.001

Race/ethnicity and state of residence remained significant predictors of behavioral intention in some models. For the heat-vulnerable model, Blacks had greater behavioral intentions compared to Whites (*b*: 0.55, *p* = 0.030), as did Hispanics (*b*: 0.77, *p* = 0.008). Individuals in Ohio or Michigan had lower behavioral intentions compared to respondents in California (*b*: −0.76, *p* = 0.019 for the heat-vulnerable model; *b*: −0.81, *p* = 0.014 for the allergy model; and *b*: −0.83, *p* = 0.018 for the asthma model), though other states did not differ.

Belief that climate change was affecting health (at post-test) was associated with greater behavioral intentions in the asthma triggers model (*b*: 0.57, *p* = 0.037).

### In-depth interviews

Multiple themes emerged regarding participants’ understanding of climate change and health based on viewing study materials.

#### Theme 1: Little differentiation between climate change and weather

The majority of respondents had only a superficial understanding of climate change. Prior to viewing any posters, all said they had heard of climate change. However, when probed to describe climate change (including as it related to health), responses revealed that day-to-day experiences with weather informed many of their concepts about climate change. Only two spontaneously mentioned the term “global warming” [34].

*“The climate change today is okay. It’s nice, it’s not too hot. So yes, you can tell the difference and it’s not too overwhelming as far as the heat climate, ‘cause I wasn’t sweating and I didn’t feel dehydrated, but I still needed to drink water. You can tell the difference as far as the climate outside.”*

-- Respondent with pollen allergies and a child with asthma

#### Theme 2: Emphasis on immediate-term health management

Respondents regarded the posters primarily as straightforward instructions for what to do to protect themselves from weather and/or environmental triggers. Respondents focused mainly on the health management aspect of the posters, citing the “health tips” as the most useful component of the posters. Most respondents had better recall and could spontaneously describe immediate-term recommendations (i.e., to deal with the short-term health effects of weather conditions) than any other information on the posters.

For example, one poster sought to connect individual food choices with health issues (obesity and heart disease) as well as carbon footprint (i.e., resource-intensive agriculture including meat production, non-local foods and processed foods). This health message used an adaptation approach to reduce individual health risk to extreme heat by lowering obesity and heart disease, while the carbon footprint message used a mitigation approach to describe individual food choices that affected the environment. Participants focused almost exclusively on the “healthy eating” message. Multiple respondents had received heart disease or obesity advice previously from their doctors, and said that the posters reinforced this advice. However, none responded to the carbon footprint components of the food posters, except to express confusion about how some of the images (e.g., a factory used to manufacture processed foods emitting air pollution) related to healthy eating.

#### Theme 3: Disconnected from collective action on climate change

Interview participants expressed little interest in longer-term climate change mitigation strategies (e.g., using less energy or voting for clean power). Two respondents touched upon systemic issues, such as food pricing or companies that pollute the air. These respondents expressed a desire for more information, but they also regarded these issues as under the control of external forces. These external forces were repeatedly spoken of as “they,” though whether “they” are government, corporations or some other social forces was unclear. Despite some interest in external, systemic factors, the posters did not help respondents make connections between individual actions and collective action.

*“You know what they should do? Raise the [prices of fast food] and lower the price of vegetables.”*

-- Respondent with asthma and obesity/diabetes

*“The air pollution caught my attention. I don’t understand it that well… It’s a big issue in America with trash and air and water – everything we need, like natural sources, they’ve just been off and they’ve just not been helpful. There’s just been problems with the oxygen in the air, the water… I found that interesting. I would like to have more information on that, the air pollution.”*

-- Respondent with pollen allergies

#### Theme 4: Real people experiencing health effects

Participants repeatedly emphasized the need for photographs of real human beings, as opposed to illustrated human figure icons or cartoons. Respondents felt that seeing real faces would be more relatable and instructive. Suggestions included dynamic images of people suffering from the health conditions described in each poster (e.g., being overheated, sneezing or having red, watery eyes from allergies) and taking some kind of protective action.

*“It should have someone that’s actually having some asthma problem. An inhaler, a nebulizer, something that shows… asthma-wise. [The current images] are just people, You don’t really see them having to do anything because of the problem. [Include] something that’s showing that it’s affecting them.”*

-- Respondent with asthma

#### Theme 5: Reaching target audiences

Participants had clear ideas about where the posters should appear. Doctor’s offices and health clinics were nearly unanimous suggestions. Another popular idea was public transportation (inside the subway and on buses, or in subway or bus stations). Offices of health and human services or social services, or transitional housing, were offered as a way to reach target audiences, as were cafeterias, fast food restaurants and grocery stores.

## Discussion

This study found that viewing concise, targeted messages about the health impacts of climate change significantly increases knowledge and beliefs about the relationship between climate change and health among chronically ill, low-SES populations. Another major finding was that greater knowledge of vulnerabilities and triggers is associated with these populations having greater intentions to practice recommended behaviors for climate change adaptation and mitigation. Believing that climate change is affecting their health or household members’ health also predicted higher behavioral intentions. Our study demonstrates that health communication materials targeting vulnerable audiences regarding climate change impacts on chronic health conditions increase knowledge and beliefs about climate change. It cannot be determined, based on the results of this study, whether these increases in knowledge and beliefs about climate change will result in sustained behavior change, but higher knowledge scores were significantly associated with positive behavioral intentions in these results. Health behavior theories describe knowledge, beliefs and behavioral intention as predictors of behavior change [35]. Previous studies have demonstrated that messages about extreme weather events, such as heat waves and hurricanes, can successfully reduce risk behaviors, morbidity and mortality associated with these events [13, 14].

Qualitative research has previously found that the public is receptive to messages about climate change with a public health frame [36], and other research has demonstrated that low-SES populations may have immediate-term, pragmatic priorities and interests regarding environmental impacts on health [18]. Our findings support both of these conclusions. Our results indicate that framing climate change as a health issue is, indeed, an effective approach for vulnerable audiences, and the most positively received content addressed individual, immediate-term health effects and practical advice for protective behaviors.

Certainty in belief that climate change is happening, as well as belief that climate change was affecting health, both increased significantly upon viewing the study materials. A substantial proportion of respondents replied that they “don’t know” whether climate change is happening, or whether it is harming their health, at baseline. The majority of movement on belief ratings was the shift from “don’t know” (at pre-test) to affirmative responses (at post-test). Previous audience segmentation research regarding climate change has categorized respondents with high levels of uncertainty and low issue involvement as “the Disengaged” [37]. The Disengaged segment has a greater proportion of racial or ethnic minorities and low-SES individuals than any of the six identified audience segments [38]. Our sample reflected these demographics as well. If the respondents who responded “don’t know” to questions about belief certainty had qualified as “Disengaged” prior to viewing the study materials, our study provides some evidence that engaging this audience segment on an issue that is immediately relevant to them – their chronic health condition – is an effective way of reaching them with climate change communication and disambiguating their beliefs about climate change.

Of particular interest is the significant improvement in knowledge that heat and carbon dioxide are allergy triggers between pre- and post-test. Respondents were largely aware at baseline that plants are allergy triggers, and a substantial proportion extrapolated that longer growing seasons for plants would trigger allergies. However, the role of heat and carbon dioxide is more distal. Higher heat and greater levels of carbon dioxide contribute to longer growing seasons and allow pollen-producing plants to flourish (a mechanism that was explained in the allergy poster). Before viewing the study materials, less than half of respondents chose heat and carbon dioxide as allergy triggers, but a significantly greater proportion correctly identified the role of these triggers at post-test. However, qualitative interviews revealed that other explanations for complex mechanisms conveyed in these materials may need improvement; the food systems poster, for example, did not resonate beyond reinforcing existing knowledge about healthy eating.

Qualitative interviews revealed that vulnerable audiences are primarily concerned with the immediate-term health impacts in the posters, and particularly protective behaviors, that affect them or their families. “Staying healthy” was a desirable goal, and adaptation appeared to resonate most with this audience. There was little interest in longer-term climate change mitigation strategies, and interview participants did not connect the role of individual behaviors to climate change mitigation, despite this information being provided by the posters. Climate change, like the weather conditions that participants used to describe their understanding of it, is the domain of systemic forces and seen as outside of their control; by contrast, personal health is within their locus of control.

The posters were effective in improving knowledge when administered online. However, both survey respondents and in-depth interview participants maintained that they would best be displayed in poster form, in locations frequented by target audiences -- doctor’s offices, public transportation, social services buildings and retail establishments.

### Limitations

This study has multiple limitations that must be considered in the design of future research that expands upon these preliminary findings. This was a study to test health communication materials of previously unknown effectiveness, not an evaluation of an intervention or a mass media campaign, and questions about generalizability remain. This study relied on a small sample of users of an online community bulletin board in multiple cities. Online surveys present several advantages, including efficient access to a geographically diverse population, targeted recruitment of a unique population (low income and chronically ill), and the reduction of response bias on a potentially controversial topic caused by a researcher’s presence [39]. Issues related to sampling were the primary disadvantage of using an online survey for this study. A link to the survey was posted on a public, regional online bulletin board and therefore it is impossible to (a) estimate response rate (i.e., how many eligible individuals viewed the recruitment advertisement and elected to respond), (b) determine the extent to which bulletin board users are representative of the target population, and (c) generate a sampling frame due to the unknown size of the community of billboard users, and what proportion of all residents of the geographic region they comprise. It is also difficult to determine whether respondents are accurately representing their demographic characteristics, although this is similarly a concern with traditional paper surveys [39]. The small sample size may not be adequately representative of the target audience, particularly within each city. Furthermore, selection bias is likely because the study did not employ random selection of respondents (or cities), and online community bulletin board users may differ from target audience members to an unknown degree (e.g., in terms of Internet access).

Recruitment ads were posted in locations that were selected according to demographic characteristics (low income and high racial/ethnic diversity) and environmental conditions (poor air quality) to ensure that local populations may resemble the target audience. Respondents were further screened individually to meet inclusion criteria for low income and low educational attainment. Additionally, the survey software allowed for screening of IP addresses by geographic region, so the validity of respondents’ physical location corresponding with desired geographic criteria was reasonably assured. Despite the small sample size, significant changes were observed in measures of knowledge and beliefs about climate change. Future research can expand this work by applying a probability sampling method in large-scale evaluations of message interventions or media campaigns to ensure representativeness and therefore generalizability. This survey employed a quasi-experimental pre-post design to measure within-subject changes, but additional studies may include a control condition to determine the extent of testing effects on increases in knowledge or beliefs.

There is a possibility of desirability bias. The recruitment announcement did not mention climate change, but it did announce that a study was being conducted among individuals with specific health conditions in their household. Respondents’ self-reporting of their household health condition(s) cannot be verified for accuracy. It is possible that, knowing only that it was a study on health conditions, respondents exaggerated their poor household health status to meet survey criteria. (Respondents were not, in fact, excluded if they replied they had none of the specified health conditions; even so, less than three percent said they had none). It is therefore possible that survey respondents are healthier than self-reported. However, the most common reported chronic condition was asthma, and levels of knowledge for asthma triggers were high at pre-test, reflecting experience with asthma that corresponds with the high prevalence of self-reported household asthma. The other area of potential desirability bias is at post-test, after respondents learned that the study was, indeed, about climate change. The increase in stated beliefs about climate change may reflect respondents’ wish to provide the “right” answer after knowing the purpose of the study [40]. However, any social desirability bias may have been tempered by the online survey format, which introduces less social pressure than an interviewer-administered survey [41]. Furthermore, the most compelling results from the study are difficult to fabricate (tests of knowledge), and other results consider the influence of varying levels of knowledge (which is less prone to bias) on behavioral intention (which is more prone to bias). If behavioral intention scores are artificially inflated, the finding could be interpreted to mean that people with more knowledge are more likely to provide biased reports of behavioral intentions. Even in this case, knowledge would be associated with a greater awareness of the desired behavior.

### Recommendations for message development

The materials used for this study were designed to be eye-catching and dynamic illustrations of the relationship between climate change and individual chronic health conditions. Colorful graphics with short text descriptors were an appropriate way to communicate these mechanisms. Based on the findings of the online survey and the qualitative interviews, we have developed recommendations for education materials that target audiences who are vulnerable to climate change health impacts due to their pre-existing health conditions and low SES [2].

#### Recommendation 1

##### Relate climate change to existing experience with health impacts

Complicated mechanisms of climate change, including distal or causative forces, can be understood if the eventual outcome is a relevant health impact. In this study, individuals with health conditions were knowledgeable about their symptoms and chronic disease management. Communication materials can refer to these experiences to engage vulnerable audiences on climate change, emphasizing the relationship between climate change and health outcomes or symptoms, rather than extensively educating the respondent about the health condition itself.

#### Recommendation 2

##### Distinguish between personal health actions and individual contributions to collective action

Despite a substantial portion of the posters being devoted to collective action toward mitigation, respondents in the qualitative portion of our study primarily focused on recommendations for individual health behaviors (adaptation). Therefore, rather than using conceptual terminology such as “Healthy You,” “Healthy Places,” and “Healthy Planet” (as our study materials did), labeling recommended actions according to their intended effect provides greater clarity (e.g., “What You Can Do For Your Health” and “What You Can Do to Prevent Climate Change”). Despite appreciating that there is a link between climate change and health, audiences still regarded the recommended actions as having separate benefits.

#### Recommendation 3

##### Use photographs of people experiencing health conditions

A consistent suggestion in qualitative interviews was to use photographs of real people experiencing health effects or symptoms that audiences would recognize and empathize with. Ideally, these photographs would also show people taking recommended action(s).

#### Recommendation 4

##### Combine improvements in knowledge with recommendations for behavior

Our study demonstrated that greater knowledge was associated with greater intentions to engage in recommended actions. Improving knowledge about climate change and health is a useful endeavor, but messages should subsequently encourage audiences to act on specific behaviors. Individual behaviors that benefit immediate-term health and safety may resonate the most with this audience, but it remains important to engage them in opportunities for collective action.

## Conclusions

Populations that are vulnerable to the health effects of climate change can benefit from communication materials that explain, using illustrations and simplified language, how climate change affects chronic health conditions and how to engage in protective health behaviors.
